# Extragnathic Giant Cell Granuloma as a Rare Consideration for a Periorbital Mass in an Infant: A Case Report

**DOI:** 10.7759/cureus.89820

**Published:** 2025-08-11

**Authors:** Gabriela N Cepeda De Jesus, Ernest F Hidalgo Cedeno, Ajay Malhotra

**Affiliations:** 1 Department of Radiology, Hartford Hospital, Hartford, USA; 2 Department of Pathology, Yale School of Medicine, New Haven, USA; 3 Department of Radiology and Biomedical Imaging, Yale School of Medicine, New Haven, USA

**Keywords:** benign lesion, giant cell granuloma, giant cell lesion, pediatric mass, zygomatic bone

## Abstract

Giant cell granulomas (GCGs) are a benign condition that commonly affects females in their first and second decades of life and almost exclusively involves the jaws. We describe a rare presentation of a GCG involving the zygomatic bone in a five-month-old infant, an exceedingly uncommon location and age group for this entity. While GCGs are most frequently seen in the mandible or maxilla of young females, to our knowledge, there are no previous reports describing zygomatic involvement in this age group. Through detailed multimodality imaging, surgical pathology, and clinical follow-up, we highlight the diagnostic considerations, recurrence potential, and role of adjuvant therapy in managing this lesion.

## Introduction

Giant cell granulomas (GCGs) are benign, occasionally locally aggressive lesions commonly involving the mandible and the maxilla [1]. The term giant cell reparative granuloma (GCRG) was first used in the 1950s by Jaffe [2]. However, it has since fallen out of favor, and its use is not recommended due to the debate on whether it truly represents a reparative process [3]. When GCGs primarily involve bone, they are referred to as central GCGs; when confined to the gingival or alveolar mucosa, they are termed peripheral GCGs [1].

The etiology of GCGs is multifactorial. Most lesions are considered reactive, possibly triggered by local trauma, inflammation, or vascular injury, although a subset demonstrates neoplastic features [4]. Recent molecular studies have identified mutually exclusive somatic mutations in TRPV4, KRAS, and FGFR1 in approximately 70% of cases [5,6]. These mutations activate the MAPK/ERK pathway, promoting cellular proliferation and osteoclastogenic signaling [6,7]. Aggressive subtypes may harbor additional genetic alterations, such as LRP1B and NOTCH4 mutations, which may contribute to their more destructive behavior [8].

Most affected patients are females, with most lesions occurring in patients < 30 years [9]. Radiographically, these lesions can be unilocular in the early stages of development, later becoming multilocular and expansile, with remodeling and thinning of the involved cortex [10].

Although histologically benign, these lesions can behave aggressively, leading to significant bone destruction and functional compromise if not promptly recognized and managed. This case illustrates the rare occurrence of a GCG in the zygomatic bone of a five-month-old infant, emphasizing its potential for recurrence. Extragnathic GCGs in infants are exceedingly rare, highlighting the importance of considering this entity in the differential diagnosis of periorbital masses.

## Case presentation

**Figure 1:**
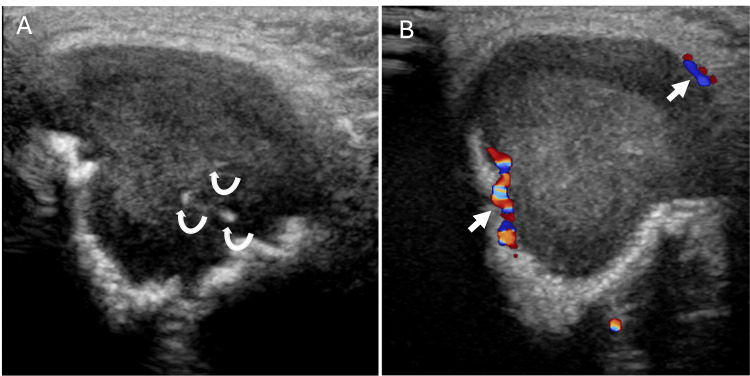
Targeted grayscale (A) and color Doppler (B) sonographic evaluation of the area of clinical concern demonstrates a solid mass with few internal punctate echogenic foci (curved arrows), likely calcifications, and minimal peripheral vascularity on color Doppler (arrows).

**Figure 2:**
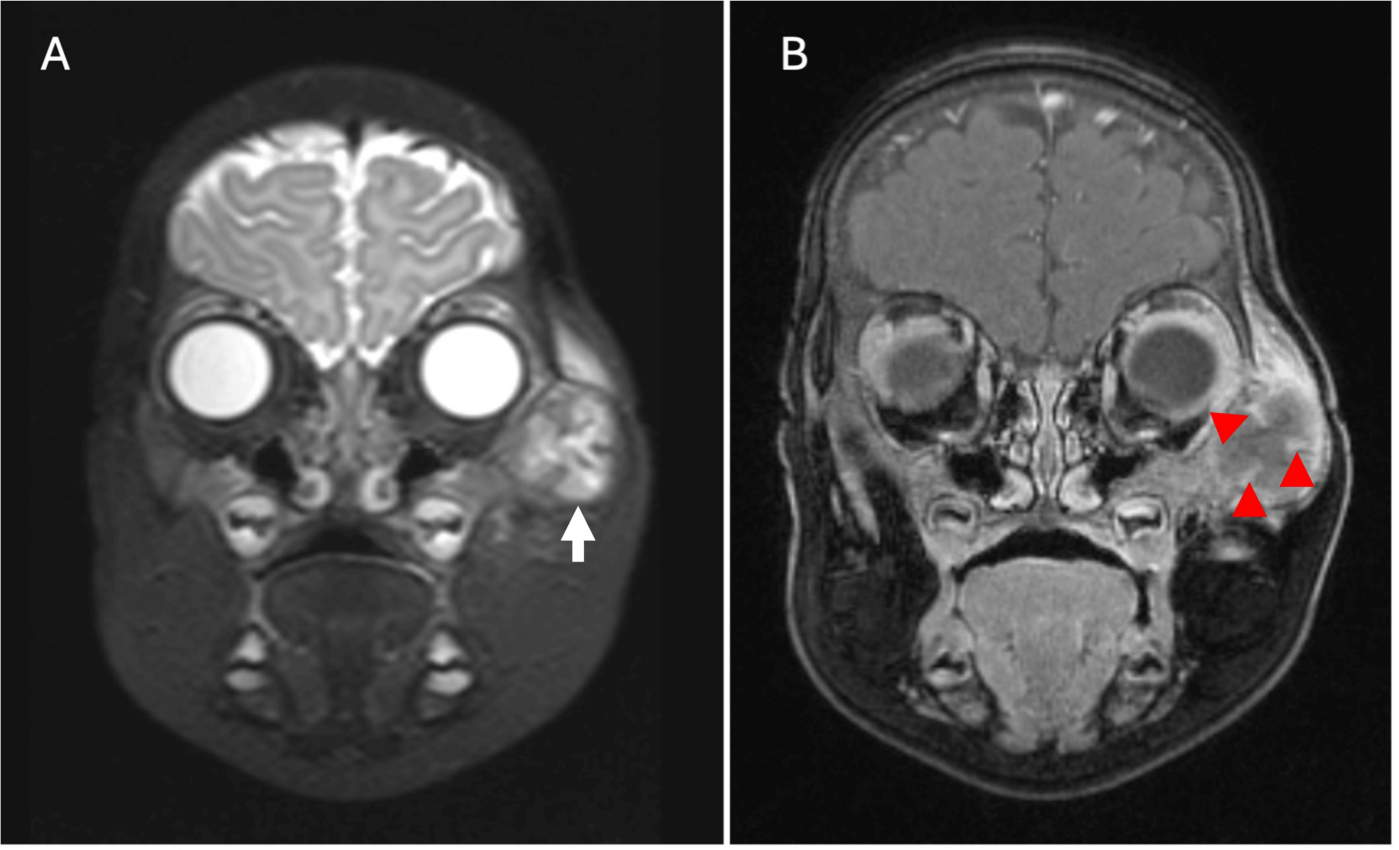
Coronal T2-weighted (A) and coronal T1-weighted post-contrast (B) images demonstrate a septated T2-hyperintense infraorbital lesion centered in the left zygomatic bone (arrow) with associated peripheral and septal enhancement (arrowheads).

**Figure 3:**
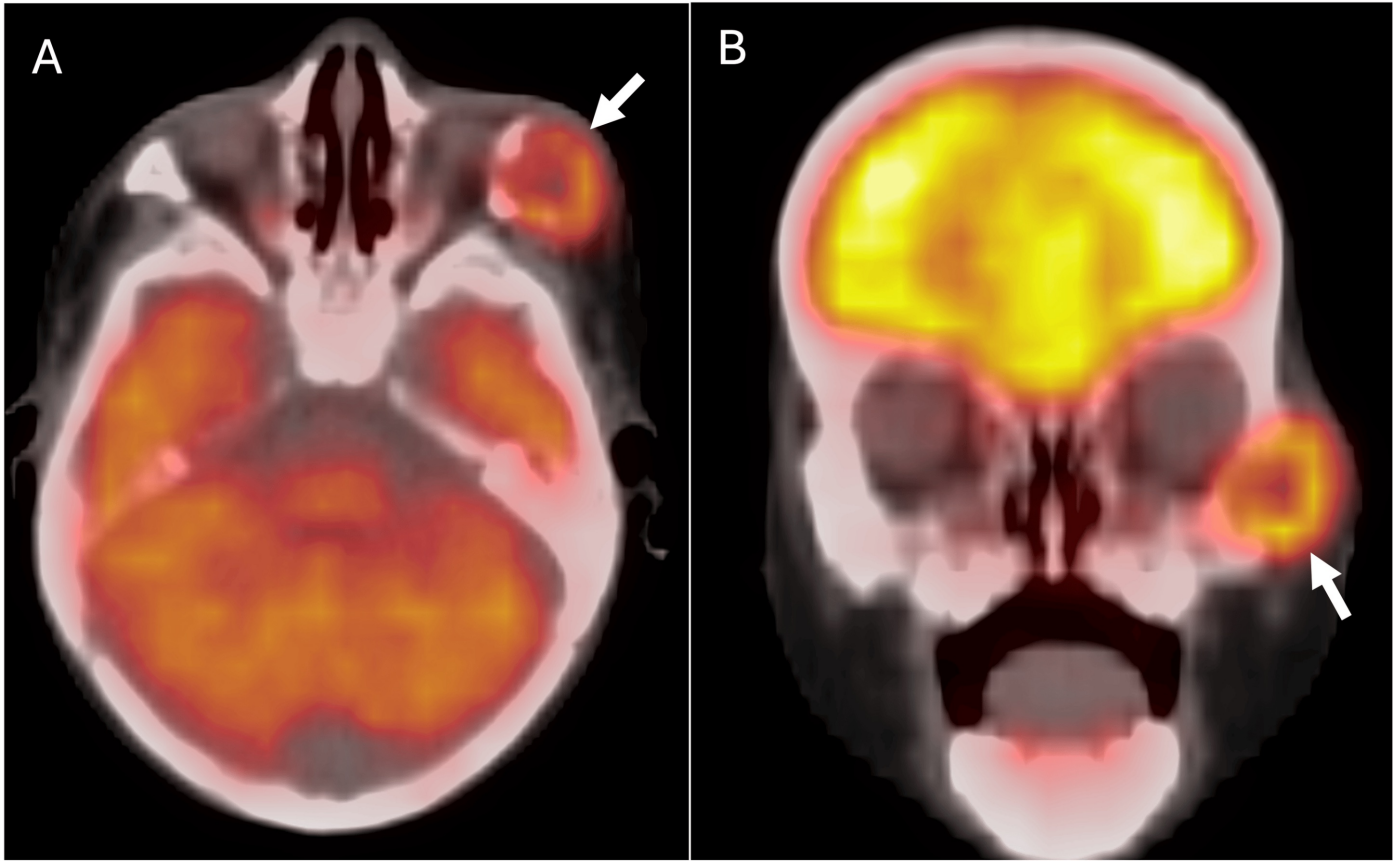
Axial (A) and coronal (B) fused FDG PET/CT images show moderate hypermetabolism (arrow) corresponding to the expansile septated left zygomatic mass.

We report the case of a five-month-old girl who presented to the pediatric clinic with an enlarging left infraorbital mass. The patient’s mother reported that the mass had been growing for the past month. A firm, non-tender, 3-cm subcutaneous mass in the left infraorbital/lateral orbital area was palpated on physical examination. A focused sonographic evaluation of the area of concern in the left infraorbital region was performed as an initial workup, demonstrating a solid-appearing lesion with a few punctate echogenic foci, likely calcifications (Figure 1). Subsequently, MRI of the face was performed on a 3T scanner using a dedicated protocol. T1-weighted images were acquired with a TR/TE (Repetition Time/Echo Time) of 595/10 ms and a slice thickness of 2.5 mm. T2-weighted short tau inversion recovery (STIR) sequences were obtained with TR/TE/TI (Inversion Time) of 3800/193/160 ms. Post-contrast images were acquired following the intravenous administration of 1.2 mL of gadoteric acid (Dotarem). MR images demonstrated a T2-hyperintense, 2.0 x 1.7 x 2.2 cm, peripherally enhancing left infraorbital mass with small septations involving the left zygomatic bone (Figure 2). A positron emission tomography (PET)/CT performed 74 minutes after an intravenous injection of 1.07 mCi (39.6 MBq) of fludeoxyglucose F 18 (F18-FDG) showed moderate hypermetabolism (SUVmax 3.9) of the left zygomatic bone mass (Figure 3), which could signify a range of conditions, including benign or malignant processes. No other hypermetabolism lesions were identified.

Laboratory evaluation revealed normal urine vanillylmandelic acid (VMA) at 8.7 mg/g creatinine (reference: 5.5-26.0 mg/g creatinine) and urine creatinine of 22 mg/dL (reference: 2-28 mg/dL). Serum calcium was within normal limits at 9.8 mg/dL (normal: 8.7-11.0 mg/dL), phosphorus was 4.7 mg/dL (normal: 4.5-6.7 mg/dL for infants), and alkaline phosphatase was 184 U/L (normal: 110-320 U/L). Serum beta-human chorionic gonadotropin (β-hCG) was undetectable (<1 mIU/mL).

**Figure 4:**
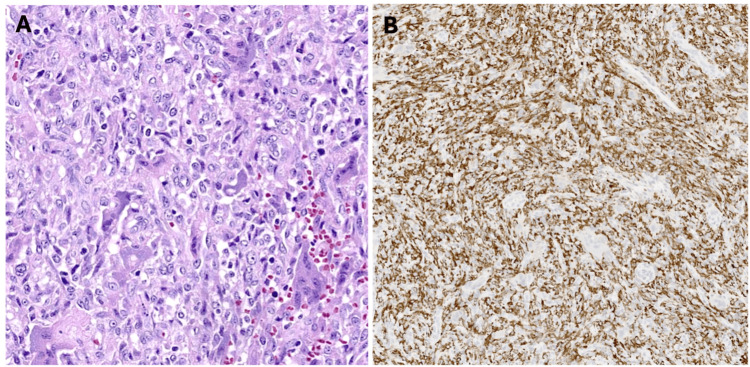
Hematoxylin and eosin (H&E) stain (A) shows a proliferation of mitotically active spindle to polygonal cells with interspersed osteoclast-like giant cells. CD163 immunostaining (B) highlights histiocytic cells.

The patient was taken to the operating room for a biopsy via a subtarsal incision. Biopsy showed a central area of necrosis with a surrounding band of mildly atypical histiocytoid tumor cells, admixed with osteoclastic giant cells and rare eosinophils, preliminarily favoring a histiocytic tumor with recurrence potential. Three weeks later, the patient underwent total resection with lateral canthoplasty. The final pathology showed similar histomorphologic findings of a heterogenous tumor with spindle to polygonal plump histiocytoid cells, with admixed osteoclast giant cells in a variably collagenous stroma, consistent with a giant cell granuloma (Figure 4).

**Figure 5:**
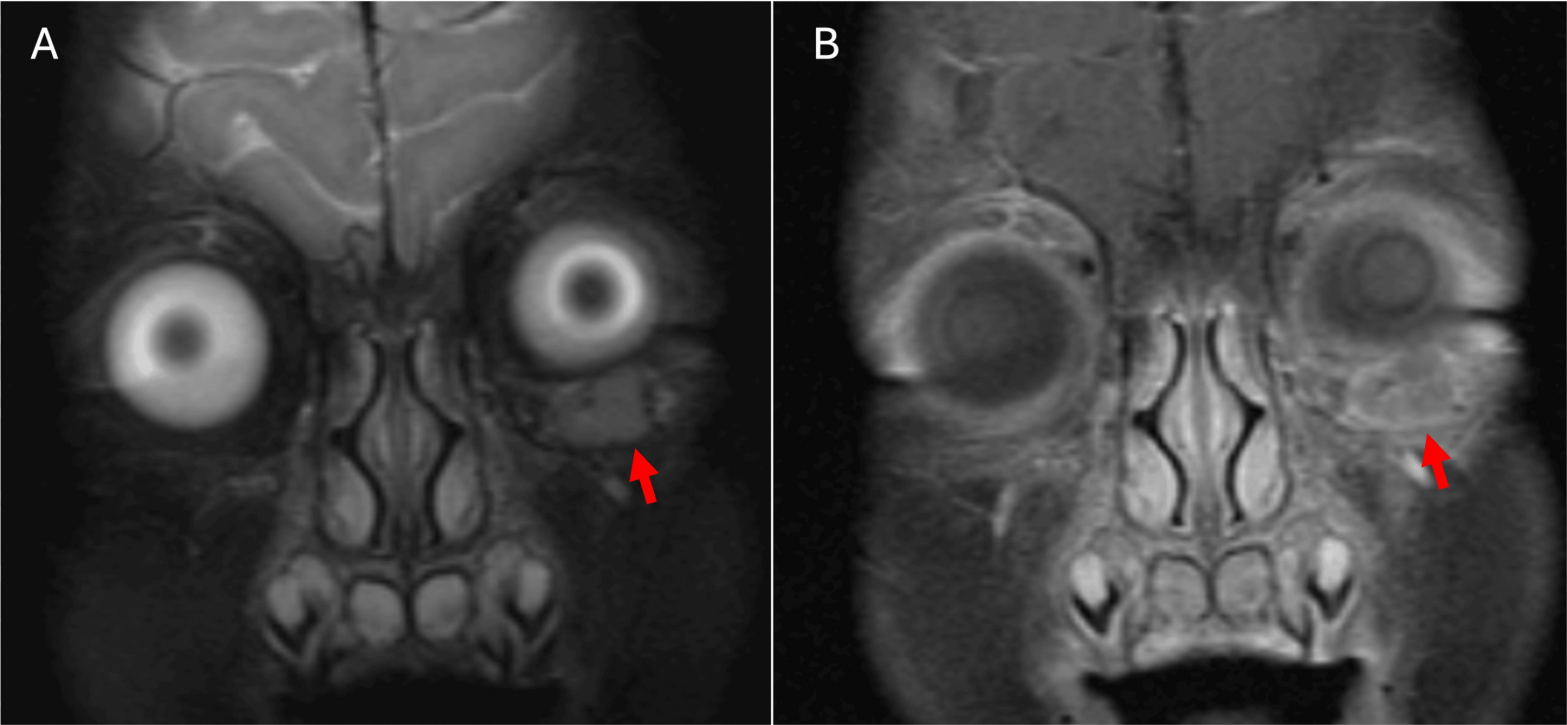
Coronal T2-weighted (A) and T1-weighted post-contrast (B) images performed seven weeks after resection demonstrate a new T2 isointense to mildly hyperintense enhancing lesion in the left inferior eyelid (arrow), which was confirmed to represent a site of recurrence upon re-resection.

Five weeks following resection, the patient’s parent noted an enlarging nodule in the left lower eyelid. MRI of the face performed seven weeks after resection demonstrated a new 1.5 x 0.8 x 1.0 cm left inferior eyelid enhancing lesion concerning for recurrence (Figure 5). The patient underwent re-resection with intralesional steroid injection (triamcinolone 20 mg (40 mg/1 mL)) intraoperatively. Pathology confirmed this represented a site of recurrence. The patient received additional adjuvant steroid injections (triamcinolone 20 mg (40 mg/1 mL)) in the left infraorbital region three weeks and five weeks after re-resection. The patient has since remained free of recurrence for five years.

## Discussion

Although almost exclusively occurring in the jaws, GCGs can rarely occur in other locations in the head and neck. To our knowledge, there have been a few cases of GCGs of the temporal and sphenoid bone [11,12], but no GCGs of the zygomatic bone have been reported. This case illustrates the importance of keeping your differential considerations open even when the disease process does not fit the usual location or patient’s demographics. GCG should be considered a differential diagnosis when an expansile lytic lesion with variable mixed solid and cystic components is seen on imaging [3].

Regardless of being benign, GCGs can exhibit aggressive local behavior and recur after resection [13], as in this case. GGCs have been reported to have a wide range of recurrences, with a recent study by Ari et al. that included 23 patients reporting a recurrence rate of 21.4% in peripheral GCG and 33.3% in central GCG [14]. Factors that contribute to the recurrence of GCGs include aggressive lesion behavior, cortical bone perforation, tooth displacement, root resorption, and the surgical approach [9,14]. The recommended clinical approach for surveillance of these lesions is regular clinical and radiographic follow-up for at least three to five years after treatment, as most recurrences occur within this period [15].

The primary treatment is surgical removal, which ranges from simple curettage to en-bloc resection [13]. In some aggressive cases, intralesional steroid or denosumab injection could be additional treatment options, as was performed in this case [13]. Alternative treatment options include radiation, calcitonin, and interferon alfa-2a (IFN-α2a) [13], though they were not recommended in this case due to the patient’s young age and numerous side effects associated with these therapies. 

The rationale for intralesional steroid therapy in the treatment of GCG is to inhibit the proliferation of fibroblasts and multinucleated giant cells, reduce local inflammation, and promote lesion regression [16,17]. Triamcinolone hexacetonide 20 mg/mL or triamcinolone acetonide 10-40 mg/mL have comparable efficacy and are typically administered as a 1:1 dilution with 2% lidocaine containing epinephrine, at a dose of 1 mL per 1 cm³ of radiolucent lesion, usually in six weekly or biweekly injections [17]. Clinical and radiographic monitoring is essential to assess response and determine if additional injections or surgical intervention are needed [17].

The primary differential diagnoses of GCG are giant cell tumors of the bone and brown tumors of hyperparathyroidism. Differentiating these lesions is difficult on the basis of imaging and microscopic features alone, and molecular profiling may be needed to achieve a definite diagnosis. In cases of suspected giant cell lesions, serum calcium and parathyroid hormone levels must be obtained to exclude hyperparathyroidism [18].

## Conclusions

This case underscores the importance of including extragnathic giant cell granuloma in the differential diagnosis of periorbital masses in infants, despite its rarity. Accurate diagnosis relies on a combination of imaging, histopathology, and clinical correlation, particularly when lesions occur in atypical locations like the zygomatic bone. While GCG is a benign entity, its potential for local recurrence necessitates close postoperative surveillance and, in some cases, adjuvant therapy. Early recognition and a multidisciplinary approach can optimize outcomes.
